# Complement factor H and interleukin gene polymorphisms in patients with non-infectious intermediate and posterior uveitis

**Published:** 2012-07-11

**Authors:** Ming-ming Yang, Timothy Y.Y. Lai, Pancy O.S. Tam, Sylvia W.Y. Chiang, Carmen K.M. Chan, Fiona O.J. Luk, Tsz-Kin Ng, Chi-Pui Pang

**Affiliations:** Department of Ophthalmology and Visual Sciences, The Chinese University of Hong Kong, Hong Kong, China

## Abstract

**Objective:**

To investigate the associations of complement factor H (*CFH*), *KIAA1109*, and interleukin-27 (*IL-27*) gene polymorphisms in patients with non-infectious intermediate and posterior uveitis.

**Methods:**

The study cohort consisted of a total of 95 Chinese non-infectious uveitis patients, including 38 patients with intermediate uveitis (IU), 38 patients with Vogt–Koyanagi–Harada disease (VKH), and 19 patients with Behçet’s disease and 308 healthy controls. The genotypes of *CFH*-rs800292, *KIAA1109*-rs4505848, and *IL27*-rs4788084 were determined using TaqMan single nucleotide polymorphism genotyping assays.

**Results:**

The frequency of carriers of G allele for *CFH*-rs800292 was significantly higher in patients with non-infectious intermediate and posterior uveitis than in controls (GG/AG versus AA; p=0.02). No significant association was found between uveitis and both *KIAA1109*-rs4505848 and *IL27*-rs4788084. In stratified analysis by gender, the frequency of carriers with G allele for *KIAA1109*-rs4505848 was significantly higher in male uveitis patients than in male controls (GG/AG versus AA; p=0.034). There was no significant difference in allelic and genotypic frequencies for *CFH*-rs800292 and *IL27*-rs4788084 in either male or female groups. In addition, higher frequency of *KIAA1109*-rs4505848 G allele was found in Behçet’s disease patients compared with controls and IU patients (p=0.01 and p=0.003, respectively).

**Conclusions:**

Our results demonstrated that *CFH*-rs800292 and *KIAA1109*-rs4505848 are associated with non-infectious intermediate and posterior uveitis. Moreover, gender susceptibility for uveitis might be involved in the *KIAA1109* gene and the *KIAA1109-*rs4505848 polymorphism might be associated with the development of Behçet’s disease.

## Introduction

Uveitis is a sight-threatening intraocular inflammatory disease and can be classified into anterior, intermediate, posterior, and panuveitis anatomically [1]. Intermediate uveitis (IU) and posterior uveitis are characterized by inflammation of the ciliary body, vitreous, retina, or choroid. They can occur in isolation or associated with other systemic immunological diseases. Some cases of IU and posterior uveitis might be secondary to ocular or systemic infections such as tuberculosis and toxoplasmosis. IU and posterior uveitis can also develop in specific ocular or systemic conditions including Vogt–Koyanagi–Harada disease (VKH), Behçet’s disease, sympathetic ophthalmia, sarcoidosis, and birdshot chorioretinopathy [2,3].

Although the exact cause of many forms of non-infectious uveitis is unclear, the pathogenesis might be related to genetic predisposition coupled with environmental factors [4]. Many uveitis-associated genes have now been identified, of which several are immune-related genes including genes for expression of interleukins and chemokines. This further strengthens the concept that endogenous immune mechanisms play important roles in the development of uveitis [5-8].

The complement system is part of the innate immune defense mechanism and is involved in modulating various immune and inflammatory responses. Recent studies have demonstrated that complement system activation is critical for the development of autoimmune uveoretinitis and suppression of the host’s complement system could completely inhibit experimental autoimmune anterior uveitis (EAAU) [9,10]. Under normal conditions, the complement system is active at a low level and is tightly regulated by various complement regulatory proteins (CRegs), such as complement factor H (CFH), decay-accelerating factor, and S-protein [11]. Disruption in the balance of complement activation and CRegs will result in harmful effects and lead to several immune-related diseases including uveitis [12,13]. CFH is one of the most important regulators in the alternative complement pathway and is involved in the pathogenesis of immunological diseases [14-16]. Recent studies suggested that variants in the *CFH* gene are associated with several immune-mediated diseases [17-19]. In addition, our previous study also demonstrated that *CFH-*rs800292 184G as a genetic risk marker for anterior uveitis in Chinese females [20].

Interleukins are potent inflammatory mediators and also known to be involved in the pathogenesis of uveitis. The levels of interleukin 2 (IL-2), interleukin 21 (IL-21), and their receptors were found to be upregulated in both experimental autoimmune uveitis (EAU) animals and in uveitis patients [21-24]. Recent studies have shown that retinal cells could suppress uveitis through interferon-gamma-mediated production of IL-27 in target tissues, while IL-27 expression was also upregulated during uveitis [25-27]. Several single nucleotide polymorphisms (SNPs) in the interleukin genes such as *IL1*, *IL10*, and *IL23R* have been found to be associated with different types of uveitis [28-30]. Recently, genome-wide association studies (GWAS) have also identified several candidate SNPs associated with immune-mediated diseases such as type 1 diabetes mellitus, rheumatoid arthritis, celiac disease, and Graves’ disease [31-33]. Some of these genetic loci could be replicated reciprocally in different diseases, suggesting that they could be general genetic risk factors for multiple autoimmune diseases [34-36].

Taking together, we hypothesize that *CFH-*rs800292, *IL-27*-rs4788084, and rs4505848 within the *KIAA1109/Testis nuclear RNA-binding protein (Tenr)/IL2/IL21* gene cluster might be involved in the pathogenesis of IU and posterior uveitis. The purpose of our study is to determine the association of these immune-associated SNPs in patients with non-infectious intermediate and posterior uveitis.

## Methods

### Study design and subjects

The study protocol was approved by an institutional review board and all procedures were conducted according to the tenets of the Declaration of Helsinki. Informed consent was obtained from all study subjects after the nature of the study was explained.

Patients were recruited in the Hong Kong Eye Hospital and all patients underwent detailed ophthalmic assessment including visual acuity testing, intraocular pressure measurement, slit-lamp and dilated fundus examinations. Clinical details were also collected including age, sex, medical history such as systemic illness including rheumatological diseases, diabetes mellitus, hypertension, and heart disease; age at initial presentation and laterality. The definition of non-infectious intermediate and posterior uveitis was based on the International Uveitis Study Group (IUSG) clinical classification [3]. Patients were categorized into three specific diagnostic groups including IU, VKH, and Behçet’s disease. All IU patients had IU in isolation without posterior uveitis or panuveitis, while VKH and Behçet’s disease patients had either panuveitis or posterior uveitis. Screening for sarcoidosis was performed in all patients. Screening for multiple sclerosis in IU patients was only performed when clinically indicated due to the low incidence of multiple sclerosis in our locality. Patients with uveitis secondary to ocular or systemic infections were excluded from the study. Three hundred and eight subjects aged 50 years or older with no evidence of eye disease except senile cataract were recruited as controls.

### DNA extraction and genotyping

Venous blood was obtained from each subject and genomic DNA was extracted with a DNA extraction kit (QIAamp; Qiagen, Hilden, Germany) according to the manufacturer’s instructions. *CFH*-rs800292, *KIAA1109*-rs4505848, and *IL27*-rs4788084 SNPs were genotyped by TaqMan allelic discrimination assay (TaqMan; Applied Biosystems [ABI], Foster City, CA) according to the manufacturer’s instructions. All PCR amplifications were performed with the following thermal cycling conditions: 95 °C for 10 min followed by 40 cycles of 92 °C for 15 s, and 62 °C for 1.5 min (rs4505848 and rs4788084); and 60 °C for 1 min (rs800292), respectively. All PCR reactions were performed with Taq polymerase (HotStarTaq Plus; Qiagen) in an automated thermal cycler (model 9700; ABI). Pre- and post-PCR plate readings were performed on a sequence detection system (Prism 7000; ABI), and the allele types were confirmed by the system software (Prism 7000 SDS software version 1.1; ABI).

### Statistical analysis

Hardy–Weinberg equilibrium (HWE) was tested by *χ^2^* test for genotype frequencies of the SNPs in control group. Allelic and genotypic frequencies between cases and controls were compared by *χ^2^* test or Fisher exact test. Dominant and recessive models in term of minor allele were applied to look for associations. Stratified analyses based on gender and specific forms of uveitis were also performed. One-way ANOVA (ANOVA) was used to compare the age of patients in different subgroups. Odds ratios (OR) and 95% confidence intervals (CI) were calculated. A p-value of <0.05 was considered statistically significant.

## Results

### Patient demographics

Ninety-five patients with non-infectious intermediate and posterior uveitis were recruited, including 38 (40.0%) patients with IU; 38 (40.0%) patients with VKH and 19 (20.0%) patients with Behçet’s disease (Table 1). Eighty-seven (91.6%) patients had bilateral uveitis, and 8 (8.4%) had unilateral involvement. There were 45 (47.4%) males and 50 (52.6%) females. All patients with Behçet’s disease had a history of oral aphthous ulcer. Five of the patients with Behçet’s disease had panuveitis and the remaining 14 had posterior uveitis including retinal vasculitis and retinitis. One patient with IU was found to have sarcoidosis. A significantly higher proportion of males was found in the Behçet’s disease group, with 78.9% compared with 34.2% and 44.7% in the IU and VKH groups, respectively (χ^2^ test, p=0.006). The mean±standard deviation (SD) age at presentation was 39.0±6.7 years for IU patients, 50.0±8.4 years for VKH patients, and 41.1±9.8 for Behçet’s disease patients. The mean age at presentation of VKH patients was significantly older than patients with IU and Behçet’s disease (one-way ANOVA Fisher LSD, p=0.001 and p=0.024).

**Table 1:** Demographic details of subjects with non-infectious intermediate or posterior uveitis.

		**Gender**		**Age (years)**	
**Uveitis type**	**No. of subjects**	**Male (%)**	**Female (%)**	**Mean**	**Range**
Intermediate uveitis (IU)	38	13 (34.2)	25 (65.8)	39.0	18–72
Vogt–Koyanagi–Harada (VKH) disease	38	17 (44.7)	21 (55.3)	50.0	23–78
Behçet’s disease (BD)	19	15 (78.9)	4 (21.1)	41.1	18–59
Total	95	46 (48.4)	50 (52.6)	43.8	18–78

### Associations between SNPs and non-infectious intermediate and posterior uveitis

The genotype frequencies of all three SNPs in control subjects conformed to the Hardy–Weinberg equilibrium. The frequency of carriers of G allele for *CFH*-rs800292 was significantly higher in uveitis patients than in controls (GG/AG versus AA; p=0.02, OR=2.74). No significant difference in the genotypic and allelic frequencies was observed for *KIAA1109*-rs4505848 and *IL27*-rs4788084 (Table 2).

**Table 2:** Comparison of genotype and allele frequencies of rs800292, rs4505848, and rs4788084 polymorphisms in patients with uveitis and control subjects.

**Polymorphism**	**Uveitis (n=95)**	**Controls (n=308)**	**p-value**	**Odds Ratio (95% CI)**
rs800292** (CFH 184G/A)**				
**Genotype**				
AA	6 (6.3)	48 (15.6)	0.068§	
AG	49 (51.6)	145 (47.1)	0.40†	
GG	40 (42.1)	115 (37.3)	0.02‡	2.74 (1.13–6.62)
**Allele**				
A	61 (32.1)	241 (39.1)	0.081	1.36 (0.96–1.92)
G	129 (67.9)	375 (60.9)		
rs4505848** (KIAA1109 A/G)**				
**Genotype**				
GG	17 (17.9)	50 (16.2)	0.49§	
AG	53 (55.8)	157 (51.0)	0.23†	
AA	25 (26.3)	101 (32.8)	0.70‡	
**Allele**				
G	87 (45.8)	257 (41.7)	0.32	
A	103 (54.2)	359 (58.3)		
rs4788084** (IL27 C/T)**				
**Genotype**				
TT	8 (8.4)	21 (6.8)	0.80§	
CT	36 (37.9)	126 (40.9)	0.81†	
CC	51 (53.7)	161 (52.3)	0.60‡*	
**Allele**				
T	52 (27.4)	168 (27.3)	0.98	
C	138 (72.6)	448 (72.7)		

Data are the number of subjects (% of the total group). § χ^2^ test for 2×3. * Fisher exact test. † p-value for dominant model. ‡ p-value for recessive model.

### Associations between SNPs and uveitis stratified by subtype of uveitis

In subgroup analysis based on uveitis subtype, the frequencies of *KIAA1109*-rs4505848 G allele and GG homozygosity were significantly higher in Behçet’s disease patients than in control subjects (p=0.01 and p=0.031, respectively; Table 3). Significantly higher frequencies of *KIAA1109*-rs4505848 G allele and GG homozygosity were also detected in patients with Behçet’s disease compared with IU (p=0.003 and p=0.011, respectively; Table 4). Similar differences however were not observed between Behçet’s disease versus VKH and IU versus VKH. For the other two SNPs (*CFH*-rs800292 and *IL27*-rs4788084), there was no significant difference in allelic and genotypic frequencies among different subgroups or compared with controls.

**Table 3:** Comparison of genotype and allele frequencies of rs800292, rs4505848, and rs4788084 polymorphisms in subgroups of noninfectious uveitis and control subjects.

**Polymorphism**	**BD (n=19)**	**IU (n=38)**	**VKH (n=38)**	**Controls (n=308)**	**p^BD-C^**	**p^IU-C^**	**p^VKH- C^**
rs800292** (CFH 184G/A)**							
**Genotype**							
AA	0 (0)	3 (7.9)	3 (7.9)	48 (15.6)	NS	NS	NS
AG	12 (63.2)	19 (50.0)	18 (47.4)	145 (47.1)			
GG	7 (36.8)	16 (42.1)	17 (44.7)	115 (37.3)			
**Allele**							
A	12 (31.6)	25 (32.9)	24 (31.6)	241 (39.1)	NS	NS	NS
G	26 (68.4)	51 (67.1)	52 (68.4)	375 (60.9)			
rs4505848** (KIAA1109 A/G)**							
**Genotype**							
GG	7 (36.8)	3 (7.9)	7 (18.4)	50 (16.2)	0.028§	NS	NS
AG	10 (52.6)	20 (52.6)	23 (60.5)	157 (51.0)	0.043†		
AA	2 (10.5)	15 (39.5)	8(21.1)	101 (32.8)	0.031‡*		
**Allele**							
G	24 (63.2)	26 (34.2)	37 (48.6)	257 (41.7)	0.01	NS	NS
A	14 (36.8)	50 (65.8)	39 (51.4)	359 (58.3)			
rs4788084** (IL27 C/T)**							
**Genotype**							
TT	1 (5.3)	4 (10.5)	3 (7.9)	21 (6.8)	NS	NS	NS
CT	9 (47.4)	14 (36.8)	13 (34.2)	126 (40.9)			
CC	9 (47.4)	20 (52.6)	22 (57.9)	161 (52.3)			
**Allele**							
T	11 (28.9)	22 (28.9)	19 (25.0)	168 (27.3)	NS	NS	NS
C	27 (61.1)	54 (71.1)	57 (75.0)	448 (72.7)			

Data are the number of subjects (% of the total group). § χ^2^ test for 2×3. * Fisher exact test. † p-value for dominant model. ‡ p-value for recessive model. NS Not Significant.

**Table 4:** Comparison of genotype and allele frequencies of rs4505848 in specific uveitis subgroups.

				**BD versus IU**		**BD versus VKH**		**IU versus VKH**	
**Polymorphism**	**BD (n=19)**	**IU (n=38)**	**VKH (n=38)**	**p**	**OR (95% CI)**	**p**	**OR (95% CI)**	**p**	**OR (95% CI)**
rs4505848** (KIAA1109 A/G)**									
**Genotype**									
GG	7 (36.8)	3 (7.9)	7 (18.4)	0.008§		NS	-	NS	-
AG	10 (52.6)	20 (52.6)	23 (60.5)	0.024†	5.54(1.12–27.54)				
AA	2 (10.5)	15 (39.5)	8 (21.1)	0.011‡*	6.81(1.51–30.59)				
**Allele**									
G	24 (63.2)	26 (34.2)	37 (48.6)	0.003	3.30(1.46–7.42)	NS	-	NS	-
A	14 (36.8)	50 (65.8)	39 (51.4)						

Data are the number of subjects (% of the total group). § χ^2^ test for 2×3. * Fisher exact test. † p-value for dominant model. ‡ p-value for recessive model. NS Not Significant.

### Associations between SNPs and non-infectious intermediate and posterior uveitis stratified by gender

In stratified analysis based on gender, the frequency of carriers of G allele for *KIAA1109*-rs4505848 was significantly higher in male uveitis patients than in male controls (GG/AG versus AA; p=0.034, OR=2.56). Similar association was not detected in females. There was no significant difference in allelic and genotypic frequencies for both *CFH*-rs800292 and *IL27*-rs4788084 in either male or female patients compared with respective control subjects (Table 5 and Table 6). Similar stratified analysis based on gender was not performed in disease subgroups due to the small sample size.

**Table 5:** Comparison of genotype and allele frequencies of rs800292, rs4505848, and rs4788084 polymorphisms in male uveitis patients and male control subjects.

**Polymorphism**	**Male uveitis patients (n=45)**	**Male controls (n=125)**	**p-value**	**Odds Ratio (95% CI)**
rs800292** (CFH 184G/A)**				
**Genotype**				
AA	3 (6.7)	17 (13.6)	0.23§	
AG	20 (44.4)	63 (50.4)	0.13†	
GG	22 (48.9)	45 (36.0)	0.29‡*	
**Allele**				
A	26 (28.9)	97 (38.8)	0.093	1.56 (0.93–2.63)
G	64 (71.1)	153 (61.2)		
rs4505848** (KIAA1109 A/G)**				
**Genotype**				
GG	10 (22.2)	23 (18.4)	0.11§	
AG	28 (62.2)	62 (49.6)	0.034†	2.56 (1.05–6.22)
AA	7 (15.5)	40 (32.0)	0.58‡	
**Allele**				
G	48 (53.3)	108 (43.2)	0.098	1.50 (0.93–2.44)
A	42 (46.7)	142 (56.8)		
rs4788084** (IL27 C/T)**				
**Genotype**				
TT	1 (2.2)	9 (7.2)	0.37§	
CT	21 (46.7)	48 (38.4)	0.70†	
CC	23 (51.1)	68 (54.4)	0.30‡*	
**Allele**				
T	23 (25.6)	66 (26.4)	0.88	
C	67 (74.4)	184 (73.6)		

Data are the number of subjects (% of the total group). § χ^2^ test for 2×3. * Fisher exact test. † p-value for dominant model. ‡ p-value for recessive model.

**Table 6:** Comparison of genotype and allele frequencies of rs800292, rs4505848, and rs4788084 polymorphisms in female uveitis patients and female control subjects.

**Polymorphism**	**Female uveitis patients (n=50)**	**Female controls (n=183)**	**p-value**	**Odds Ratio (95% CI)**
rs800292** (CFH 184G/A)**				
**Genotype**				
AA	3 (6.0)	31 (16.9)	0.095§	
AG	29 (58.0)	82 (44.8)	0.77†	
GG	18 (36.0)	70 (38.3)	0.052‡	3.20 (0.93–10.93)
**Allele**				
A	35 (35.0)	144 (39.3)	0.43	
G	65 (65.0)	222 (60.7)		
rs4505848** (KIAA1109 A/G)**				
**Genotype**				
GG	7 (4.0)	27 (14.8)	0.94§	
AG	25 (50.0)	95 (51.9)	0.72†	
AA	18 (36.0)	61 (33.3)	0.89‡	
**Allele**				
G	39 (39.0)	149 (40.7)	0.76	
A	61 (61.0)	217 (59.3)		
rs4788084** (IL27 C/T)**				
**Genotype**				
TT	7 (14.0)	12 (6.6)	0.11§	
CT	15 (30.0)	78 (42.6)	0.52†	
CC	28 (56.0)	93 (50.8)	0.14‡*	
**Allele**				
T	29 (29.0)	102 (27.9)	0.82	
C	71 (71.0)	264 (72.1)		

Data are the number of subjects (% of the total group). § χ^2^ test for 2×3. * Fisher exact test. † p-value for dominant model. ‡ p-value for recessive model.

## Discussion

In this study, we investigated the association of three immune-related SNPs in the *CFH*, *KIAA1109*, and *IL27* genes with non-infectious intermediate and posterior uveitis. Our results demonstrated that *CFH*-rs800292 and *KIAA1109*-rs4505848 were significantly associated with IU and posterior uveitis, and in particular Behçet’s disease. Moreover, different gender-susceptibility to the genetic association was also identified.

The *CFH* gene is located in the long arm of chromosome 1 (1q32), which is a major soluble inhibitor of the alternative complement cascade [13]. Activated complement due to loss of CRegs regulation by CFH might cause self-tissue damage in sensitive organs like the eyes [11]. In vivo studies have revealed that human RPE cells can synthesize and express CFH, and upregulated secretion of CFH by RPE can suppress the development of EAU [12,37,38]. In our previous studies, we have found that polymorphisms in the *CFH* gene are associated with the development of neovascular age-related macular degeneration (AMD) as well as anterior uveitis in females [20,39]. In addition, *CFH* has also been found to be associated with other immune-mediated diseases such as multifocal choroiditis, hemolytic-uremic syndrome (HUS) and glomerulonephritis [17,19]. In this study, *CFH*-rs800292 was found to be associated with non-infectious intermediate and posterior uveitis, which showed a recessive effect (GG/AG versus AA; p=0.02, OR=2.74). This finding is consistent with our previous study on AU and *CFH*-rs800292 [20]. Although the previously shown gender-susceptibility in female AU patients was not found, there was a trend toward higher 184G allele frequency for *CFH-*rs800292 in female patients with intermediate and posterior uveitis compared with female controls (p=0.052). The discrepancy might be related to the small sample size or distinct pathogenesis other than AU and further studies to investigate the gender-susceptibility involvement in IU and posterior uveitis are required. Nonetheless, these findings strengthen the concept that complement system especially CFH is involved in the development of uveitis. The I62V variant (rs800292) is located in one of the regulatory domains and the amino acid substitution might lead to structural changes affecting the ability of C3b binding and thus reducing the activation of the alternative pathway C3-convertase (C3bBb) [40,41]. This subsequently leads to excessive activation of the complement system and might induce immunologic disorders. The exact mechanism is still unclear and further studies are required to investigate the functional interaction of CFH with uveitis.

SNP rs4505848 is located in the region encompassing *KIAA1109/Tenr/IL2/IL21* in chromosome 4q27. *IL2* and *IL21* genes are both functional candidates for autoimmune diseases as they may be involved in the regulation of T-cells responses. The levels of IL2, IL21, and their receptors were found to be significantly elevated in uveitis patients and animal uveitis models [21-23]. Both Th1 and Th17 effector cells have been shown to independently induce uveitis in animal models [42]. Functional studies have revealed that Th17 cells contribute to uveitis through expanded IL-2, while IL-21 was highly expressed and promoted the differentiation of Th17 cells in both in vitro and in vivo studies [23,25]. In our present study, stratified analysis demonstrated that SNP rs4505848 was significantly associated with IU and posterior uveitis in male patients. Such association was not found in our previous AU study [20]. The gender specificity for rs4505848 might account for different pathway of T-cell response in posterior uveitis. It might also be related to the small number of subjects; the wide variety of uveitis syndromes; or the distinct complex regulatory mechanism for autoimmune diseases. These findings suggest that the region especially the *IL-2* and *IL-21* genes may have important roles in the development of non-infectious intermediate and posterior uveitis. These results are consistent with studies on *IL2* and Behçet’s disease [43] and support the concept that non-infectious posterior uveitis is predominantly mediated by T-cell response. Therefore, the use of anti-IL-2 therapy in various forms of uveitis might be a promising treatment option [44].

IL-27 is a cytokine within the IL6/IL12 family and consists of EBI3 and p28 subunits. Studies in EAU have demonstrated that IL-27 is constitutively expressed in retinal ganglion cells and photoreceptors. IL-27 can promote Th1 but inhibit Th17 cells differentiation, which causes mutual antagonism between the two pathways [25]. In our study, no association was found between the *IL-27* SNP and IU and posterior uveitis, even stratified by gender or disease subgroups.

Our study contains several limitations. First, the relatively small sample size, particularly in the subgroup analyses, will reduce the statistical power of the study and therefore some modest associations could not be detected. Selection bias might also occur in our study but this is unlikely to be significant since the patients recruited in this study are likely to be representative of our clinic population. We have recruited around 30% of the 300 patients with non-infectious intermediate, posterior and panuveitis who have attended the uveitis clinic during the recruitment period. In addition, some of the P values would no longer be statistically significant after adjusted for multiple testing. Finally, we have only evaluated three selected immune-related SNPs in this study and thus our findings will not reflect the disease risk of unexamined variants in these genes. This might result in us missing some genetic variants associated with intermediate and/or posterior uveitis and further evaluation of these genes by direct sequencing to uncover more variants will be beneficial to identify variants with relevant function in uveitis.

In conclusion, we identified the associations of *CFH*-rs800292 and *KIAA1109*-rs4505848 with non-infectious intermediate and posterior uveitis in Chinese patients. Some different gender-specific susceptibility might also be involved. Further studies replicating the candidate SNPs in other ethnic groups and to determining the biologic roles of these polymorphisms in uveitis are warranted.
